# Individual identity and affective valence in marmoset calls: in vivo brain imaging with vocal sound playback

**DOI:** 10.1007/s10071-018-1169-z

**Published:** 2018-02-27

**Authors:** Masaki Kato, Chihiro Yokoyama, Akihiro Kawasaki, Chiho Takeda, Taku Koike, Hirotaka Onoe, Atsushi Iriki

**Affiliations:** 1 Laboratory for Symbolic Cognitive Development, RIKEN Brain Science Institute, Wako, Saitama Japan; 2 Division of Bio-Function Dynamics Imaging, RIKEN Center for Life Science Technologies, Kobe, Hyogo Japan; 3 RIKEN-NTU Research Centre for Human Biology, Lee Kong Chian School of Medicine, Nanyang Technological University, Singapore, Singapore; 4 Present Address: Research Development Section, Research Promotion Hub, Office for Enhancing Institutional Capacity, Hokkaido University, Sapporo, Hokkaido Japan

**Keywords:** [^18^F] Fluorodeoxyglucose positron emission tomography, Vocal perception, Nonhuman primate, Individual discrimination, Emotion

## Abstract

As with humans, vocal communication is an important social tool for nonhuman primates. Common marmosets (*Callithrix jacchus*) often produce whistle-like ‘phee’ calls when they are visually separated from conspecifics. The neural processes specific to phee call perception, however, are largely unknown, despite the possibility that these processes involve social information. Here, we examined behavioral and whole-brain mapping evidence regarding the detection of individual conspecific phee calls using an audio playback procedure. Phee calls evoked sound exploratory responses when the caller changed, indicating that marmosets can discriminate between caller identities. Positron emission tomography with [^18^F] fluorodeoxyglucose revealed that perception of phee calls from a single subject was associated with activity in the dorsolateral prefrontal, medial prefrontal, orbitofrontal cortices, and the amygdala. These findings suggest that these regions are implicated in cognitive and affective processing of salient social information. However, phee calls from multiple subjects induced brain activation in only some of these regions, such as the dorsolateral prefrontal cortex. We also found distinctive brain deactivation and functional connectivity associated with phee call perception depending on the caller change. According to changes in pupillary size, phee calls from a single subject induced a higher arousal level compared with those from multiple subjects. These results suggest that marmoset phee calls convey information about individual identity and affective valence depending on the consistency or variability of the caller. Based on the flexible perception of the call based on individual recognition, humans and marmosets may share some neural mechanisms underlying conspecific vocal perception.

## Introduction

Vocalization is an important tool for communication in both common marmosets (*Callithrix jacchus*) and humans. Marmosets emit a number of distinctive calls, which elicit differential behavioral responses in conspecific listeners (Epple 1968; Norcross et al. 1994; Yamaguchi et al. 2009; Watson and Caldwell 2010). Thus, particular marmoset calls appear to convey specific information. Human perception of species-specific vocalizations consists of three informational processes: (1) establishing the caller identity, (2) processing affective information, and (3) processing linguistic information with higher-order semantic content (Belin et al. 2004). Human neuroimaging has revealed species-specific voice-sensitive areas including the superior, middle and anterior temporal regions, and the limbic system including the medial prefrontal cortex and amygdala, which are associated with these processes (Dolan et al. 2001; Belin et al. 2004; Giraud et al. 2004; Formisano et al. 2008). Marmoset vocalization is likely to involve the former two processes (i.e., those with nonlanguage contents).

The long-distance contact call (i.e., a whistle-like phee call) is one of the most investigated calls in the marmoset vocal repertoire as a communication signal (Norcross and Newman 1993, 1997; Miller and Wang 2006; Chen et al. 2009; Miller et al. 2010b; Miller and Thomas 2012; Takahashi et al. 2013; Choi et al. 2015; Miller et al. 2015; Takahashi et al. 2017). However, the neural processes specific to phee call perception are largely unknown, despite the possibility that these processes involve emotional and/or social information. Unlike some other marmoset calls, phee calls are highly tonal and stereotyped (Miller et al. 2010b) and their acoustic features show inter-individual differences with stability over time, which can alter depending on arousal level and/or social context (Jones et al. 1993; Miller et al. 2010b). The phee call is frequently emitted and reciprocally exchanged between visually separated animals in a process known as antiphonal calling (Miller and Wang 2006). It has been shown that marmosets can recognize individuals from their phee calls, using antiphonal calling behavior as a metric (Miller and Thomas 2012). This ‘turn-taking’ vocal oscillation is similar to that observed during human conversation (Takahashi et al. 2013), and contingent parental vocal feedback promotes infant vocal development (Takahashi et al. 2017). Phee calls that are repeated during isolation are thought to serve to reunite the group, and have different acoustic features compared with phee calls produced while socially engaged (Norcross and Newman 1993). Marmosets can discriminate context and change their vocalization behavior in response to hearing phee calls (Chen et al. 2009; Choi et al. 2015). These results suggest that phee calls likely invoke context-dependent responses across multiple brain regions. However, electrophysiological and neuroimaging studies have mainly focused on the auditory cortex and other social calls (‘twitters’; Wang et al. 1995; Nagarajan et al. 2002; Kajikawa et al. 2008) or conspecific calls not limited to phee calls (Sadagopan et al. 2015). Only a few studies have looked beyond the auditory cortex when examining neural responses to phee calls, but these have been restricted to certain regions of interest (Miller et al. 2010a). Brain-wide analysis using phee calls associated with different social situations would fill these gaps in current knowledge.

In this study, we sought to characterize the whole-brain neural responses of phee call perception in marmosets. First, we measured behavior to confirm that listeners were able to discriminate the caller identities from only audio information by using a familiarization-discrimination playback procedure (Weiss et al. 2001; Fitch and Hauser 2004; Kaneko and Tomonaga 2008). If marmosets have the ability to hear individual differences between phee calls, we expected that their search behavior would be exaggerated when they perceived the caller change (Weiss et al. 2001; Kaneko and Tomonaga 2008). Then, we used ^18^F-fluorodeoxyglucose positron emission tomography (FDG-PET) to verify the map of regional neural activation elicited by hearing two types of stimuli involving phee calls: repeated phee calls from a single unchanging caller (single subject phee calls: SSP) and phee calls from multiple callers, where the identity of the caller changed for each call (multiple subject phee calls: MSP). We also included trials with no-auditory playback as a control condition. These three trial types provided different levels of the number of callers, as indicated by the totality of sequential calls. By comparing the metabolic activity elicited in these trials, we sought to examine brain areas related to the processing of caller discrimination. The probable regions involved include the temporal cortex and the limbic system, which are known voice-sensitive and socioemotional processing areas in primate species, including humans (Dolan et al. 2001; Belin et al. 2004; Gil-da-Costa et al. 2006; Formisano et al. 2008; Petkov et al. 2008; Perrodin et al. 2011). Finally, we measured pupillary responses in animals as they listened to two types of phee call stimuli (SSP and MSP, as for the PET experiment above) in an additional experiment. We expected differences in the pupillary response to support the differences in brain activation between the two trial types. These steps enabled us to examine neural activation elicited by auditory phee call signals from single versus multiple callers.

## Materials and methods

### Subjects

We used eight male common marmosets (*C. jacchus*) that were 3–5 years of age at the start of the behavioral and imaging experiments. The marmosets were housed in the RIKEN Center for Life Science Technology (CLST), and all had undergone either one or two PET scans in the awake state for other experiments, which had been performed at least 1 year prior to the present experiment. We used a separate group of eight similarly aged male marmosets to generate an averaged anatomical template of the common marmoset brain (see *PET data analysis*) (Yokoyama et al. 2013). We recorded phee calls from six additional male marmosets to use as playback stimuli. These animals were housed in the RIKEN Brain Science Institute (BSI) (see *Acoustic stimuli*). Animals were housed in pairs or individually in breeding rooms with a 12-h light–dark cycle (light 08:00–20:00) in both the RIKEN CLST and BSI. Each enclosure had several wooden perches, a food tray, and a water dispenser. Twice daily, the animals received solid food (CMS-1, CLEA Japan, Inc., Tokyo, Japan) mixed with an appropriate amount of powdered milk formula, honey, gluconic acid, calcium, vitamin C, and lactobacillus probiotic, supplemented with chopped boiled eggs or bananas once per week. The temperature and humidity in the breeding room were maintained at approximately 28 °C and 50%, respectively. This study complied with the current laws regarding ethical treatment of research animals in Japan, including the Act on Welfare and Management of Animals.

### *Acoustic stimuli*

The phee calls used as playback stimuli were recorded in a sound-attenuated room using SASLab (Avisoft Bioacoustics, Glienicke, Germany). The calls were recorded from six animals housed in RIKEN BSI, which is distinct from RIKEN CLST (the institute that housed the animals used in the behavioral testing and PET experiment). After giving the animals time to acclimate to the behavior chamber (60 × 60 × 60 cm), we recorded vocalizations. We then extracted the normalized phee calls from the audio files using SASLab. The sequential pattern of the playback sound stimuli for testing individual discrimination is described in the section entitled *Behavioral testing individual discrimination*. To map the brain activity specifically associated with caller identification, we used two different sound sequences described in *PET scans* and *Behavioral testing emotional arousal.*

### Behavioral testing individual discrimination

**Fig. 1:**
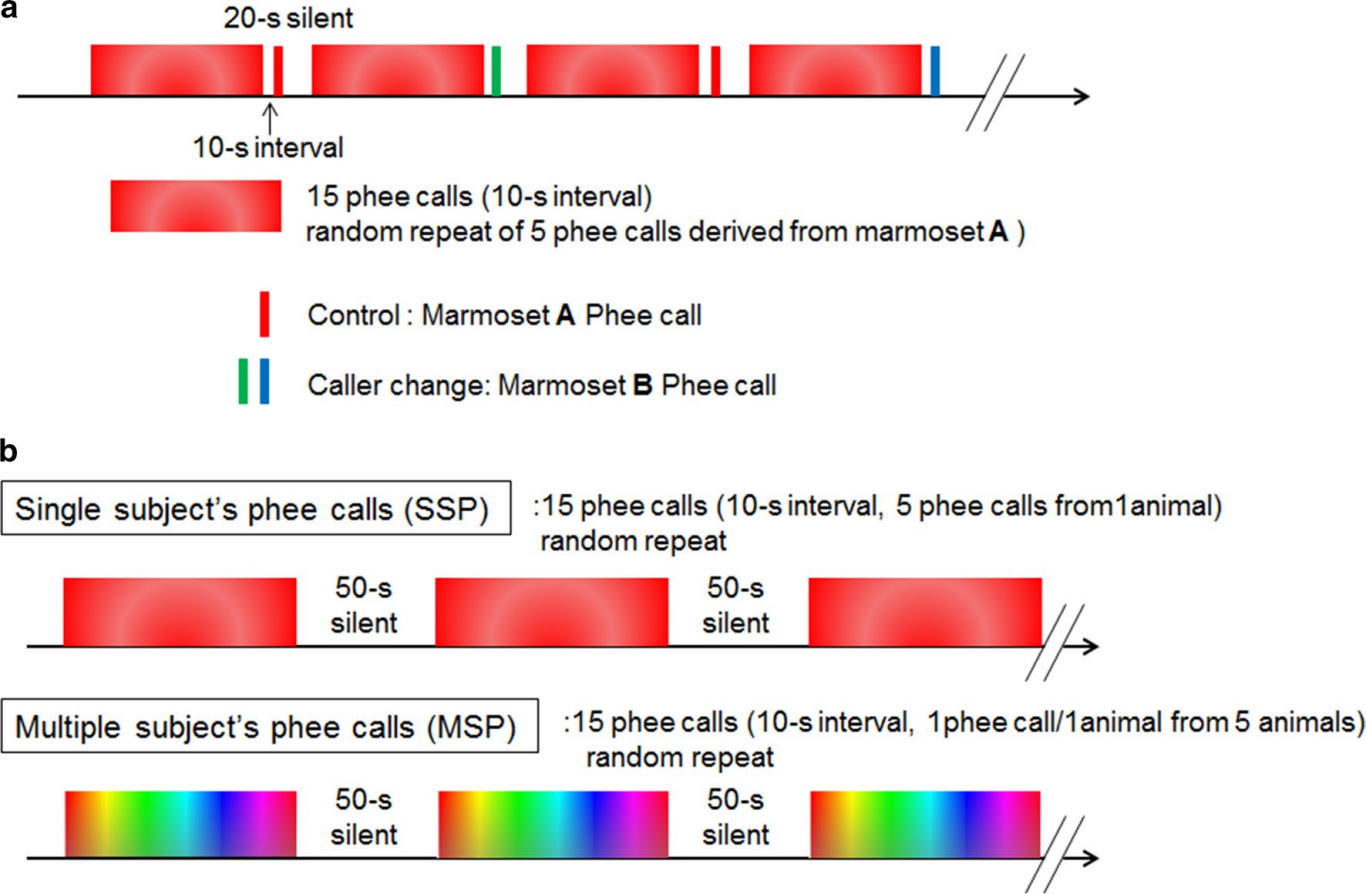
Playback paradigm. To test individual discrimination, we presented 15 consecutive randomly selected phee calls, shown in red–white gradient columns, which were each one of five phee calls recorded from marmoset *A*. Each call was separated by 10 s. After the last 10 s period, we presented a phee call from either the same individual (marmoset *A*: control stimuli, a red bar) or a different individual (marmoset *B*: caller change stimuli, a green or a blue bar) as the test stimuli (**a**). To measure perception of phee calls based on individual discrimination ability, we used two types of sound stimuli; phee calls from a single subject (SSP), shown in red–white gradient columns, and those from multiple subjects (MSP), shown in rainbow gradient columns (**b**)

We evaluated the ability of eight marmosets to perform individual caller discrimination by exposing them to phee calls using a familiarization-discrimination playback procedure. For each test session, a pair of cage-mates was separated and placed in individual cages (W472 × D280 × H285 mm) in a sound-attenuated room (W1742 × D1742 × H1900 cm). The two speakers (KS-1HQM; Kripton Co., Ltd, Tokyo, Japan) for sound playback were placed side by side facing the testing cages, and a video camera (HDC-HS300; Panasonic Corporation, Tokyo, Japan) was also placed between the speakers facing the testing cages to record animals’ responses. The sound stimuli were presented from both speakers, positioned side by side, 15 cm apart. After five 30-min acclimatization sessions in the testing environment on different days, we initiated behavioral testing using a playback procedure with the *Acoustic stimuli* at 70 dB. The sequential pattern of the playback sound stimuli is described in Fig. 1a. The sound stimuli for familiarization consisted of 15 consecutive randomly selected marmoset phee calls recorded from the same individual, which were separated by a 10-s interval. After a final 10-s interval, we presented a probe phee call for the test stimulus from either a different marmoset or from the same marmoset, with the duration of each phee call varying from 1.19 to 2.18 s. We used phee calls recorded from two marmosets in a set of 6 trials, and then, we used phee calls recorded from two marmosets different from the first set in a second set of 6 trials on another day. In each set, the different caller trials and the same caller trials were repeated alternately three times. We scored the behavioral responses on video images following the six ‘different caller’ and six ‘same caller’ trials. We focused on sound source searching behaviors from the start of phee call test stimulus presentation to 1 s after the end of phee call presentation, which were defined as the following behavior items: head turning toward speaker, body orienting toward speaker, movement toward speaker, and vocal response. One observer scored all trials and a second condition-naïve observer scored half of the trials from video images the same way as the first observer to ensure the reliability of scoring. The inter-observer reliability was 0.71, assessed using Cohen’s Kappa test (Cohen 1960), which indicates good agreement (Watkins and Pacheco 2000). Therefore, we adopted the all scores by first observer for this study. We compared the rate of response between the different caller and same caller probes using a paired *t* test. Data were expressed as mean ± standard error of the mean.

### PET scans

Four of the animals from the behavioral tests were subjected to PET scans. These animals had previously been acclimated to PET scanning conditions through three dummy scans and two PET scans that were part of other experiments. To maintain a secure head position during the PET scan, a small acrylic headpiece (diameter 10 mm; height 8 mm) had previously been aseptically attached to the surface of the skull under anesthesia (Yokoyama et al. 2010). We conducted PET scans (microPET Focus 220; Siemens Medical Solutions, Inc., Knoxville, TN, USA) according to a previously published method (Yokoyama et al. 2010). After cannulation into a tail vein, awake animals were fixed via a head holder in a sitting position with the scanner tilted to 45°. After taking a 30-min transmission scan with a ^68^Ge–^68^Ga pin source for attenuation correction, we delivered a bolus injection of FDG averaging 173.3 ± 17.2 MBq/kg through the cannula and performed scans for 90 min. To map the brain activity specifically associated with caller identification and caller situations, we used two different sound sequences that included phee calls (i.e., phee call conditions): single subject phee calls (SSP) and multiple subject phee calls (MSP), which are described in Fig. 1b. Stimuli were presented from a speaker at 70 dB. For SSP, we presented eight sequences in which groups of five calls were randomly drawn from a group of 15 calls from a single caller and presented in 10-s intervals for a total of 30 min with 50-s pauses. For MSP, we presented eight sequences in which five calls were randomly drawn from a group of 15 calls from five different callers and presented in 10-s intervals for a total of 30 min with 50-s pauses. All subjects also underwent PET scanning without auditory stimuli (i.e., control condition). Even in the control condition, we observed a noise level of 63 dB caused by electric fans or other machines in the PET scanning room. The SSP, MSP, and control (no-auditory playback) conditions for each animal were spaced at 2-week intervals and ordered randomly. The video camera (HDC-HS300; Panasonic Corporation, Tokyo, Japan) was set in the front lower side of the tilted scanner, enabling us to monitor and record the facial expressions and vocalizations of the animal. Vocalization rarely occurred in the scanner during the auditory playback and control conditions, with the exception of one animal that emitted two phee calls during SSP and one phee call during MSP, and another animal that emitted one phee call during SSP. Facial movement was scored on the videotape as the aggregated time duration of eye blinking and movements of the ears and mouth. A repeated measures one-way analysis of variance (ANOVA) revealed that the scores showed no significant differences between the SSP, MSP, and no-voice trials (*F*_2,12_ = 1.4, *p* = 0.28). The score was used as a covariate to regress out the facial movement in the statistics for PET data analysis.

### PET data analysis

**Fig. 2:**
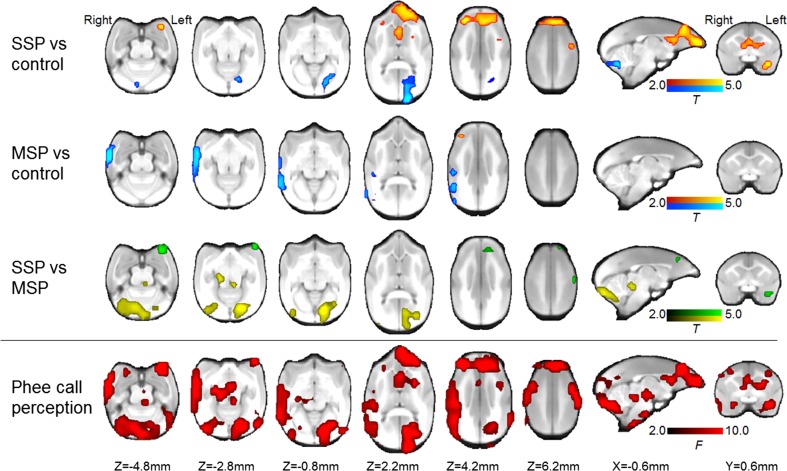
Brain maps of phee call perception. In the two top rows depicting brain slices, red–yellow colors indicate regions with statistically significant activation while listening to a series of phee calls (i.e., those from a single caller: SSP > control, those from multiple callers: MSP > control) and blue–light blue colors indicate regions with statistically significant deactivation (i.e., SSP < control, MSP < control). Green and yellow colors in the third row indicate regions with statistically significant differences between SSP and MSP (green; SSP > MSP, yellow; SSP < MSP). Red colors in the fourth row indicate regions with a statistically significant change among SSP, MSP, and control conditions with the *F*-statistic. The coordinates refer to the standard MRI of the common marmoset brain prepared in our laboratory; the coordinates are referenced to the anterior commissure on the ac–pc plane. The clusters show the statistical significance at *p* < 0.005. Colored bars are for *T* scores in the three top rows and for *F* scores in the fourth row

**Fig. 3:**
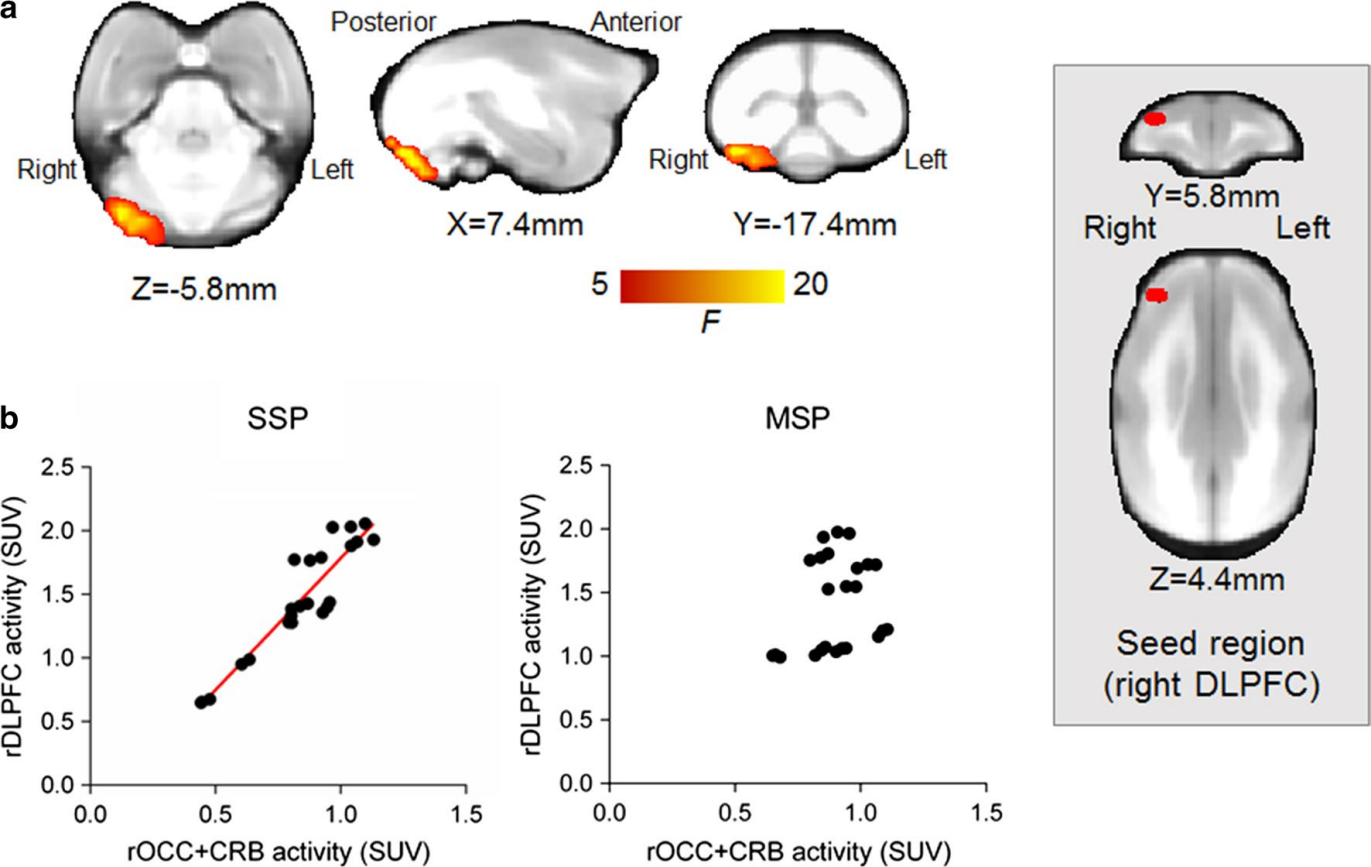
Functional connectivity map affected by different types of phee call stimuli. The red–yellow color indicates a trend toward a statistically significant interaction between the different phee call conditions in terms of the seed region activity (*F*-statistic, *p* < 0.1) (**a**). Scatter graphs represent standard uptake values (SUV) from FDG-PET data in two regions, such as the right dorsolateral prefrontal cortex (rDLPFC, seed ROI) and the occipital cortex adjoining the cerebellum (rOCC + CRB, cluster revealed by voxel–based analysis) (**b**). We found a significant correlation in the SSP but not the MSP condition (see text). The box on the right encloses the seed region in red. The coordinates refer to the standard MRI of the marmoset brain

We used the FDG images taken 60–90 min after the start of scanning as an index of local neural activity during the first ~ 30 min of PET scanning (i.e., the period of auditory exposure; Holschneider and Maarek 2004). We performed reconstruction using a filtered back-projection algorithm and projection data that had been subjected to attenuation correction via spatial smoothing of the transmission scan with a 0.5-mm Hanning filter. For statistical analysis, the reconstructed PET images (voxel size 0.38 × 0.38 × 0.796 mm), averaged over 10 min, were registered with the magnetic resonance imaging (MRI) images from each subject via rigid transformation with 1.0-mm resampling. This was done using PMOD version 3.5 (PMOD Technologies, Zurich, Switzerland), which is a software package used to analyze biomedical images. Manually descaled MRI images of individual brains were spatially normalized to the anatomic marmoset brain template by brain normalization using PMOD. The transformation matrix for each subject was applied to an MRI template from the co-registered PET images. The voxel-based statistics of the PET images were found using FEAT (FMRI Expert Analysis Tool, Version 5.98), which is available via FSL 5.0 (FMRIB’s Software Library, https://fsl.fmrib.ox.ac.uk/fsl/fslwiki). Though PET image analysis could not be directly comparable to fMRI, the voxel size in this study was 1/50 of that in previous human fMRI studies of voice perception (Belin et al. 2000; Andics et al. 2014). The approximate marmoset brain size is 1/150 of the human brain, while the thickness of cerebral cortex is about 1/2 that of humans. To map the brain activation induced by phee call playback trials, we adopted a repeated measures one-way ANOVA with trial type (SSP, MSP, no-voice) as the factor, followed by *T*-contrasts of SSP and MSP with the no-voice trials, and the contrasts between the SSP and MSP trials. The whole-brain FDG uptake value was used as a covariate to normalize for global variation. Some images showed high FDG uptake in the temporal muscle contiguous to the brain surface. To address this, we used the uptake values in the regions of interest overlapping the temporal muscle as a covariate to remove possible nearby spillover and muscle activity-related effects. To regress out facial movement, we used the score of video-taped observations as a covariate in the statistics. Voxel-based calculations were based on the nonparametric permutation method (https://fsl.fmrib.ox.ac.uk/fsl/fslwiki/Randomise) (Winkler et al. 2014). The statistical significance threshold was set at *p* < 0.005 with family-wise error rate correction following threshold-free cluster enhancement. Then, we studied functional connectivity of the right dorsolateral prefrontal cortex as a seed region of interest (ROI), because the preceding ANOVA revealed significant cluster activation in this region associated with phee call perception in both the SSP and MSP conditions (see Fig. 2, Table 1). The activity of the seed ROI (*R*), the phee call conditions (*P*), and their interaction (*R* × *P*) were entered into the design matrix. We sought to assess whether the contrast of the interaction between SSP and MSP, which represents the functional connectivity of the seed ROI, was affected by caller change in a series of phee calls. We set the *F*-statistical significance at *p* < 0.1 and the *t* statistical significance at *p* < 0.05 with family-wise error rate correction following threshold-free cluster enhancement. We also conducted an ROI-based analysis for the relation of FDG-PET radioactivity in the seed ROI to that in the cluster revealed by voxel-based analysis (Fig. 3). The activity value was represented as the standard uptake value (SUV), a value that is normalized to the body weight and dose received by the animal, in the ROIs from the FDG-PET data. The strength of the association of neural activity between the ROIs was evaluated by Spearman’s correlation coefficient. To construct the marmoset anatomic brain template, we used MRI images of eight different animals obtained with a 3-Tesla MRI scanner (Magnetom Allegra; Siemens, Erlangen, Germany), as prepared in our previous experiments (Yokoyama et al. 2013).

**Table 1:** Brain activation while hearing different types of phee call stimuli

Regions^a^	Cluster size (mm^3^)	Peak *T*^b^	Peak *x*, *y*, *z* standard space (mm)^c^
SSP > control			
Right dorsolateral prefrontal cortex, bilateral medial prefrontal cortex, left orbitofrontal cortex, the septum and the nucleus accumbens	248	7.01	5.4, 5.6, 4.2
Left somatosensory cortex	11	4.64	− 5.8, − 1.4, − 5.2
Left amygdala	10	5.50	− 5.6, 1.6, − 5.8
SSP < control			
Left occipital cortex	115	5.92	− 3.6, − 20.4, 2.2
Cerebellar vermis	36	4.98	− 0.6, − 15.4, 6.8
Right cerebellar hemisphere	13	4.71	7.4, − 14.4, − 7.8
MSP > control			
Right dorsolateral prefrontal cortex	2	6.36	6.4, 5.6, 4.2
MSP < control			
Right temporo-parietal cortex including TE, TEO, FST, TPt and adjacent occipital cortex	189	6.07	10.4, − 4.4, − 4.8
SSP > MSP			
Left piriform cortex	27	5.74	− 7.6, 3.6, − 3.8
Left medial prefrontal cortex	15	4.77	− 2.6, 6.6, 5.2
Left inferior parietal cortex (PFG)	9	5.43	− 7.6, − 3.4, 5.2
SSP < MSP			
Bilateral occipital cortex and adjacent cerebellum	459	6.41	− 3.6, − 17.4, − 2.8
Right medial geniculate nucleus	17	3.17	3.4, − 7.4, − 2.8
Left inferior colliculus	14	3.04	− 1.6, 9.4, − 3.8

^a^Anatomic locations with abbreviations in parentheses refer to a stereotaxic atlas (Paxinos et al. 2011)

^b^Statistical significance at *p* < 0.005 with family-wise error rate correction following threshold-free cluster enhancement

^c^The coordinates refer to standard MRI of the common marmoset brain prepared in our laboratory; reference is to the anterior commissure on the ac–pc plane

### Behavioral testing emotional arousal

Pupil dilation is an index of physiological, emotional, and attentional responses (Bradley et al. 2008; Ebitz et al. 2014; McGinley et al. 2015; Vinck et al. 2015). After the PET experiment, we examined the differential emotional impact of hearing sequences of phee calls (i.e., SSP and MSP) by measuring pupillary diameter. The same eight marmosets from the familiarization-discrimination procedure were used. The data from one animal were omitted because the eyes were closed for the majority of the experiment. The animals were positioned in a custom-made chair, which was modified from that used in the PET experiment to allow the marmosets to stand while maintaining a stable head position, and placed in the sound attenuation room in which the test subjects had been previously acclimated. SSP and MSP tests were spaced by an interval of at least 2 weeks and ordered randomly. The SSP and MSP stimuli used were the same as in the PET experiment. Pupillary diameter of the right eye was recorded at 90 Hz using a ViewPoint Eye Tracker system (Arrington Research, Scottsdale, AZ, USA) equipped with an infrared camera. The eye tracker measured pupil diameter in arbitrary units that were normalized with respect to the width of the window that displayed the video image of the eye. We analyzed variation in pupillary diameter using the following procedure: (1) remove samples deviating from the median by more than the standard deviation of the diameter or the vertical or horizontal aspect ratios as outliers for each trial, (2) detect variation *f(t)* of time t by setting the median as zero, subtract the median of the diameter for each trial from the sample value, (3) calculate the sum *S* of the definite integral values in which each of positive variations is time-integrated throughout the 30-min trial as follows:

$$S = \mathop \sum \limits_{i = 0}^{{\left( {m - 1} \right)/2}} \mathop \int_{{t_{2i} }}^{{t_{2i + 1} }} f\left( t \right){\text{d}}t,$$

where *t*_0_, $t_{1} , \ldots , t_{m}$ (*m* is an odd number) are the roots of the equation *f(t)* = 0.

For statistical analysis, paired *t* tests were used to compare the raw pupil diameter and S, the sum of the definite integral values of positive variation between SSP and MSP. Data were expressed as mean ± standard error of the mean.

## Results

### Behavioral response

Animals exhibited sound source searching behaviors more frequently in response to phee calls from different subjects compared with those from the same subject. We found a significant difference in the rate of occurrence of behavioral responses between probe calls from the same caller and those from different callers (*N* = 8, same caller = 22.92 ± 8.87, different caller = 68.75 ± 11.55, *t*(7) = 6.07, *p* = 0.0005), suggesting that marmosets can discriminate changes in individual marmoset phee calls.

### Brain imaging

We found a significant change in widespread brain activity associated with hearing different types of phee call sequences (see the fourth row in Fig. 2). Significant *T*-contrasts of SSP and MSP in no-voice trials are shown in Fig. 2 and Table 1. SSP is a series of phee calls repeated from a single caller, and MSP is a series of phee calls repeated from multiple callers, with a different animal producing every call. We found that, compared with the control condition, SSP induced brain activation in the right dorsolateral prefrontal, the left orbitofrontal, the bilateral medial prefrontal, and the left somatosensory cortices, and additional subcortical regions such as the bilateral septum, ventral striatum, and the left amygdala. Compared with the control condition, MSP induced brain activation in the right dorsolateral prefrontal cortex. We also found less brain activation during SSP in the left occipital cortex and the cerebellum, while less activation occurred in the right inferior parietal and temporal cortices during MSP, compared with the control condition.

When we subtracted the MSP from the SSP activation, we found activity in the left medial prefrontal cortex, the left intraparietal cortex, and the amygdala, as well as the left piriform cortex just anterior to the amygdala. All of these regions were located in or near the clusters observed when the control condition was subtracted from the SSP activation. When we subtracted the SSP from the MSP activation, we found activity in the midbrain nuclei, which are part of the early stage auditory pathway, as well as the inferior colliculus, medial geniculate nucleus, occipital cortex, and cerebellum. All of these regions were located in clusters when the SSP condition was subtracted from the control condition.

Voxel-based analysis revealed a trend toward significant functional connectivity between the right dorsolateral prefrontal cortex and the right occipital cortex adjoining the cerebellum. This connectivity was affected by the caller change in the phee call series (Fig. 3a, Table 2). The cluster was identical to a significant cluster (statistical significance *p* < 0.05) in the SSP − MSP contrast (Table 2). An inter-regional correlation with ROI-based calculations revealed that neural activity in the right dorsolateral prefrontal cortex was tightly coupled with that in the right occipital cortex adjoining the cerebellum in the SSP but not MSP condition (Fig. 3b) and the levels of significance were 0.93, *p* < 0.0001 in SSP, and 0.33, *p* = 0.11 in MSP, respectively.

**Table 2:** Functional connectivity of the right prefrontal cortex between different types of phee call stimuli

Regions^a^	Cluster size (mm^3^)	Peak statistics	Peak *x*, *y*, *z* standard space (mm)^d^
SSP versus MSP			
Right occipital cortex and adjacent cerebellum^b^	33	31.1	7.4, − 17.4, − 5.8
*SSP* > *MSP*			
Right occipital cortex and adjacent cerebellum^c^	37	5.58	7.4, − 17.4, − 5.8
*SSP* < *MSP*			
None	–	–	–

^a^Anatomic locations refer to a stereotaxic atlas (Paxinos et al. 2011)

^b^Significance at *p* < 0.1 with family-wise error rate correction following threshold-free cluster enhancement and peak statistic shown in *F* score

^c^Significance at *p* < 0.05 with family-wise error rate correction following threshold-free cluster enhancement and peak statistic shown in *T* score

^d^The coordinates refer to standard MRI of the common marmoset brain prepared in our laboratory; reference is to the anterior commissure on the ac–pc plane

### Pupillary diameter

The median of raw pupil diameter in arbitrary units was not different between the SSP and MSP conditions (SSP = 0.1026 ± 0.0054, MSP = 0.1030 ± 0.0063, *t*(6) = 0.41, *p* = 0.69). Positive variation of pupillary size, as an index of autonomic and cognitive arousal, differed between the SSP and MSP conditions, as in the PET experiment. SSP evoked a significantly greater pupillary size compared with MSP (*N* = 7, SSP = 4.81 ± 0.60, MSP = 2.96 ± 0.19, *t*(6) = 2.57, *p* = 0.042). The SSP signals may have been more salient to the listeners than the MSP signals; for instance, they could have communicated that the caller was isolated from other individuals.

## Discussion

Marmosets appear to discriminate the difference of caller when listening to conspecific phee calls. The brain regions activated by single subject phee calls (SSP) imply extensive cognitive and emotional processes and are partly reminiscent of human brain activity elicited by listening to a human voice (Dolan et al. 2001; Belin et al. 2004; Formisano et al. 2008). Multiple subject phee calls (MSP) induced brain activation in some of the SSP-activated areas. SSP and MSP induced different patterns of brain deactivation and functional connectivity. Pupillary size response, as a measure of physiological responses, indicated higher arousal levels with SSP compared with MSP. These results suggest that a series of phee calls can have multiple meanings with different emotional valence depending on the consistency or variability of the caller.

### Discriminating individuals using phee calls

Marmosets appear to discriminate between individuals according to phee calls, which corresponds to the result of a previous study using antiphonal calling (Miller and Thomas 2012). Miller and Thomas (2012) generated playback stimuli by setting calls to occur with a timing scheme that mimicked antiphonal calling. By contrast, in the current study, we used a fixed interval of 10 s and did not examine antiphonal calling. Thus, we anticipated a sound exploratory response when hearing a conspecific call (Weiss et al. 2001; Kaneko and Tomonaga 2008). The exaggerated search behavior elicited by phee calls appears likely to be due to dishabituation following habituation (Weiss et al. 2001; Fitch and Hauser 2004; Kaneko and Tomonaga 2008). In other words, marmosets show less search behavior in response to a probe call from the same subject, via a habituation effect. However, the PET and pupillometry experiments revealed that single subject phee calls (SSP) elicited stronger neural activation and a higher arousal state than multiple subject phee calls (MSP). This discrepancy might depend on the difference in the playback sound stimuli and the target phenomenon to be measured (e.g., active searching versus passive processing). During passive processing of the stimuli in which all calls came from either the same animal, or different animals, the marmosets were not surprised by a sudden change in caller identity. The other difference to the prior study is that we used phee calls for baseline and probe stimuli recorded from unfamiliar animals living in a different facility. The previous study reported that marmosets can discriminate individuals even if they are the same sex and familiar conspecifics. Our results indicated marmosets can discriminate same sex and unfamiliar conspecific calls as well. This may be related to individual differences in the acoustic features of phee calls (Jones et al. 1993; Miller et al. 2010b).

### Mapping marmoset brain activation during phee call playback

To our knowledge, this is the first neuroimaging report to address phee call perception in the awake marmoset using a whole-brain approach. SSP induced a specific pattern of brain activation, associated with salient information related to both cognitive and affective processes. The observed activation in the dorsolateral and medial prefrontal cortices and the amygdala implies increased processing of social information. This is related to human studies that examined the neural correlates of social trait judgments using human voice stimuli (Hensel et al. 2015). The orbitofrontal cortex, as well as the dorsolateral and medial prefrontal cortices and the amygdala, are involved in general affective processing such as that for fear and disgust within social information in humans (Adolphs et al. 1998; Adolphs 2013). The septum and the ventral striatum are well known to be involved in learning and expression of contextual fear conditioning in rodents (Bradfield and McNally 2010; Reis et al. 2010), as well as humans (Sheehan et al. 2004; Klucken et al. 2009). Conversely, parietal somatosensory cortex activation induced by SSP is rather difficult to interpret. Although we cannot deny the possibility of false positive activation, this may be related to the distribution of auditory frequency representations over brain regions such as the parietal somatosensory cortex, although their functional roles remain to be addressed (Pérez-Bellido et al. 2017).

We found that some of the brain activation induced by MSP was also observed during SSP. Specifically, both stimuli activated the dorsolateral prefrontal cortex, which could be related to higher cognitive processing, implying auditory attention and/or online monitoring of individual calls in working memory (Huang et al. 2013; Scott and Mishkin 2016; Wegrzyn et al. 2017). Although we might have expected MSP to elicit greater brain activation compared with SSP because of the increase in the amount of information (i.e., multiple individuals), the results show the opposite. When we subtracted MSP from SSP activation, we found activity in the medial prefrontal and intraparietal cortices, as well as in a region expanding from the amygdala to the piriform cortex. This indicates a higher arousal level during SSP versus MSP, consistent with our measure of arousal via pupillary diameter change. However, we found that sections of the subcortical auditory pathway, such as the inferior colliculus and medial geniculate nucleus, remained after subtracting SSP from MSP. This suggests that early feature-based analysis of acoustic information would be more distinguishable in MSP than in SSP, though there was no difference in high-level fine processing in the auditory cortex. We also found the occipital cortex and the adjacent cerebellum remained after subtracting SSP from MSP, similar to after subtracting SSP from control, indicating that SSP induced less activation in these areas compared with MSP. The visual- and motor-related neural processing loads are unlikely due to these results, because the experimental setting certainly fixed the head and body of the animal where they were well acclimated. Facial movement in the video-taped observation scores showed no difference, and this was regressed out by using a covariate. The functional significance of the areas activated more in MSP than in SSP is unclear, but at least it serves to indicate the different perception of the two types of phee calls. These results suggest that marmosets may discriminate the compositional properties of SSP, perceiving the repeated phee calls as being from a single isolated caller, and MSP as being from different callers in a communicative situation. Thus, both the caller situation and caller identity may be important. Marmosets use context-dependent phee calls with different acoustic features (Norcross and Newman 1993, 1997; Norcross et al. 1999). Our findings indicate that listeners may be able to receive information about the caller situation by both the compositional and acoustic features of a call sequence.

We found brain areas showing less activation during phee call playback compared with no-auditory playback, which was different between the SSP and MSP conditions, i.e., the medial occipital cortex and cerebellum were less activated during SSP, while the parietal-temporal cortex was less activated during MSP. These results might be linked with a previous report that indicated that scenic emotional stimuli induce brain deactivation during fearful and happy scenes in humans (Radua et al. 2014). Although the interpretation of brain deactivation by emotional stimuli remains uncertain (Kober et al. 2008), differential less activated brain areas by SSP and MSP compared to the control condition indicates that distinct emotional processing is occurring. However, it is paradoxical that we found less activation during MSP compared to control in the parietal-temporal cortex, which is close to the voice area in humans (Belin et al. 2000, 2004), but does not correspond with the species-specific voice area in nonhuman primates, namely the anterior temporal lobe (Petkov et al. 2008, 2009; Sadagopan et al. 2015). These results may be related to the experimental paradigm that we used of no-auditory playback including the noise of electric machines as a background rather than a noncall auditory stimulus as used in previous studies (Poremba et al. 2004; Petkov et al. 2008, 2009). In order to verify this possibility, we need to undertake further studies using a control stimulus, such as a no-call auditory stimulus.

The auditory phee call sequence in this study induced no significant activation in the auditory cortex including voice-sensitive areas in humans and macaques (Belin et al. 2000; Formisano et al. 2008; Petkov et al. 2008, 2009). It is possible that the phee calls evoked auditory processing below the statistical threshold. We expect this was not dependent on sound amplitude or familiarity with the caller because the phee calls that we used were all normalized and recorded from conspecifics that were unfamiliar to the listeners and living in a different facility. We used FDG-PET, which, unlike functional MRI, is less sensitive for detection of changes within a short time series synchronized to each call and reveals metabolic accumulation as a whole over several min. It may also be associated with the setting of the control condition as no-auditory playback including a noise. Furthermore, the unique acoustics of marmoset phee calls are highly tonal, very stereotyped, and thus highly predictable (Miller et al. 2010b). Using various calls including phee, twitter, trill, tsik, alarm, chatter, and scream, FDG-PET analysis with the same procedure in our laboratory identified general brain activation patterns for auditory processing in response to conspecific calls (activated regions included the right primary auditory cortex and the bilateral auditory association cortices, such as the anterior and middle superior temporal sulcus) (Kato et al. 2012). A previous functional MRI study of conspecific auditory perception using anesthetized marmosets used various kinds of marmoset calls as conspecific vocalizations, and reported bilateral auditory cortical activation (Sadagopan et al. 2015). The repetition associated with the similarity of the reoccurring phee calls may have contained less acoustic feature information compared with various calls stimuli.

Our functional connectivity analysis indicated that the cerebello-prefrontal circuit was specifically recruited by hearing phee calls from a single subject, suggesting that listeners may have a special neural response to a signal from SSP. Recent human neuroimaging and clinical studies have revealed that the cerebellum modulates not only motor but also cognitive and affective functions involved in linguistic processing (Stoodley 2012; De Smet et al. 2013; Mariën et al. 2014; Argyropoulos 2016). In the current study, animals did not show any difference in vocal or facial expression behavior between scan conditions; therefore, this neural network involving the cerebellum is unlikely due to motor function. Interestingly, the cerebello-prefrontal circuit is claimed to be involved in sequence processing in a working memory buffer (Mandolesi et al. 2001; Molinari et al. 2008; Stoodley et al. 2012). The cerebello-prefrontal circuit may thus be involved in higher (nonmotor) functions such as sequential prediction of conspecific calls in the specific process of SSP.

### Physiological response to phee call sequence

We hypothesized that SSP and MSP would induce differential physiological responses in terms of pupillary size because our PET data indicated that the marmosets processed cognitive and emotional information more intensely upon hearing SSP versus MSP. As expected, pupillary variation was larger during SSP compared with MSP stimuli. Pupillary size under constant luminance is an index of autonomic and cognitive arousal, regulated by norepinephrine and acetylcholine, which optimizes the decision-making process and contributes to sympathetic nervous tone in response to threats (Bradley et al. 2008; Gilzenrat et al. 2010; Eldar et al. 2013; Ebitz et al. 2014; McGinley et al. 2015; Vinck et al. 2015; Reimer et al. 2016). This finding supports our brain mapping results and suggests that marmosets hearing SSP may perceive the caller to be in an unusual situation of survival threat in contrast with MSP.

### Communication call perception in nonhuman primates

According to our results, phee calls made by a single caller are likely to be more critical than phee calls made by multiple callers for conspecific listeners, relying on their ability of individual discrimination. Individual recognition in animals has been studied by using playback calls in several social contexts such as territoriality, competition, mate selection and parental care, which are thought to reduce the cost associated with inter- and intra-group agonistic behaviors and improve reproductive success (Cheney and Seyfarth 1980; Delgado 2006; Tibbetts and Dale 2007). However, our results are unlikely to apply to any of them; rather, they seem to suggest their group ties with that general meaning, not individually specialized for individual-specific pair, family or group bonds. The contexts of marmosets making phee calls are not associated with inter- or intra-group competition. Instead, a phee call is used for affiliative communication signals between conspecifics, making ‘turn-taking’ vocal oscillation and serving to reunite the group (Norcross and Newman 1993; Miller and Wang 2006; Takahashi et al. 2013). In the current study, we used playback calls that were recorded from unfamiliar and same sex (male) conspecifics; therefore, there was no heterosexual or kin relationship between callers and listeners. From an ecological point of view, it might be strange and surprising for marmosets to hear all unfamiliar males chorusing, but our results showing MSP to be less attended to than SSP did not support that. They might be able to generalize the group ties for an unfamiliar caller beyond their personal experiences. Although marmosets are not as closely related to humans as chimpanzees or macaques, they show strong social learning skills and cognitive flexibility. This could be related to their tendency to engage in cooperative breeding, like humans (Burkart and van Schaik 2010; Vaesen 2012). In the same way, individual recognition expanded to out-of-personal relationships in marmosets may depend on their expanded breeding system.

Previous studies of animal voice perception have indicated the role of emotional and referential signaling, including individual recognition in nonhuman primates and other social organisms (Cheney and Seyfarth 1980; Seyfarth and Cheney 2003; Delgado 2006; Tibbetts and Dale 2007; Miller and Thomas 2012). However, they say nothing about the neural basis underlying it. We have provided here neurobiological evidence of conspecific call perception in the brain, which has been argued in these studies. Our neuroimaging results give new insights into voice preferring networks extending to nonauditory areas in addition to the voice areas (Belin et al. 2004; Poremba et al. 2004; Petkov et al. 2008; Andics et al. 2014). To study the biological basis of voice-related processing further, we should identify neural characteristics in clusters revealed by neuroimaging, e.g., the medial prefrontal cortex, the amygdala, the dorsolateral prefrontal cortex and the cerebellum, as systematic evidence of voice cells in the temporal pole has been provided following functional MRI experiments in monkeys (Perrodin et al. 2011). We also need further studies that consider familiarity and sex differences using multimodal neuroimaging techniques targeting the whole brain, including functional MRI in behaving marmosets during auditory tasks.
